# Risk factors for revision surgery due to construct failure after instrumented treatment of pyogenic spondylodiscitis

**DOI:** 10.1016/j.bas.2026.106283

**Published:** 2026-08-12

**Authors:** Bilal Younes, Dorothee Mielke, Charlotte Flüh, Veit Rohde, Tammam Abboud

**Affiliations:** a Department of Neurosurgery, University Medical Center Göttingen, Robert-Koch-Straße 40, Göttingen, 37075, Germany; b Department of Neurosurgery, University Hospital Augsburg, Stenglinstraße 2, Augsburg, 86156, Germany

**Keywords:** Pyogenic spondylodiscitis, Construct failure, Revision surgery, Prediction and risk factors, Spondylodiscitis, Posterior instrumentation, 360° fusion

## Abstract

**Objective:**

Construct failure following instrumented treatment for pyogenic spondylodiscitis remains a significant clinical challenge and is often associated with persistent pain or neurological deterioration requiring revision surgery. This study aimed to identify risk factors for construct failure requiring revision surgery after instrumented treatment of pyogenic spondylodiscitis.

**Methods:**

This retrospective single-center cohort study included 355 patients who underwent dorsal spinal instrumentation with or without anterior reconstruction for pyogenic spondylodiscitis at the University Medical Center Göttingen between 2013 and 2022. Construct failure requiring revision surgery was defined as the primary endpoint. Time-to-event analyses were performed using Kaplan–Meier survival estimates and Cox proportional hazards regression models. Baseline predictors included age, Charlson Comorbidity Index (CCI), preoperative C-reactive protein (CRP), and osteoporosis. An extended model additionally incorporated postoperative relapse infection and wound infection.

**Results:**

During a mean follow-up of 27 ± 9 months, 48 of 355 patients (13.8%) underwent revision surgery due to construct failure. Revision-free survival was 86.9% at 1 year, 82.7% at 2 years, and 80.9% at 3 years. In the baseline Cox proportional hazards model, a higher CCI (HR 1.08 per point increase, 95% CI 1.00–1.16; p = 0.048) and elevated CRP (HR 1.004 per mg/L increase, 95% CI 1.001–1.007; p = 0.018) were independently associated with an increased hazard of revision surgery due to construct failure.

In the extended model, relapse infection emerged as the strongest predictor of revision surgery due to construct failure (HR 11.54, 95% CI 5.05–26.36; p < 0.001), while CCI and preoperative CRP remained significant. Age, osteoporosis, and wound infection were not independently associated with revision surgery due to construct failure.

**Conclusion:**

Greater comorbidity burden and elevated preoperative CRP were associated with an increased risk of revision surgery due to construct failure, whereas postoperative relapse infection was strongly associated to revision surgery. Optimized risk stratification and stringent infection control may help to reduce revision risk.

## Highlights

• In 355 surgically treated pyogenic spondylodiscitis patients, 13.8% required revision for construct failure during a mean 27-month follow-up.
• Revision-free survival was 86.9% at 1 year, 82.7% at 2 years, and 80.9% at 3 years.
• Relapse infection was the strongest independent predictor of construct failure (HR 11.54, p < 0.001).
• Higher Charlson Comorbidity Index and elevated preoperative CRP were independently associated with increased revision risk.
• Age, osteoporosis, and postoperative wound infection were not independent predictors in multivariable analysis.

## 1 Introduction

Pyogenic spondylodiscitis is a severe spinal infection with a rising incidence across Europe. Population-based data from several European countries, including Germany and the Scandinavian region, indicate a marked increase over the past two decades, likely reflecting demographic aging, higher comorbidity burden, improved imaging techniques, and increased survival of multimorbid patients. In Germany alone, the incidence has doubled from 5.4 to 11.0 cases per 100,000 inhabitants within two decades. At hospital admission, up to 50% of patients present with neurological deficits secondary to vertebral destruction or spinal epidural abscess (Younes et al., 2026a; Thavarajasingam et al., 2023). In selected early-stage cases without instability or neurological compromise, conservative treatment with targeted antibiotic therapy, analgesia, and immobilization may be sufficient (Pluemer et al., 2023; Valancius et al., 2013; Kramer et al., 2025). However, several European cohort studies have reported improved outcomes with early surgical intervention in appropriately selected patients, including reduced mortality and shorter hospital stay (Neuhoff et al., 2025a, 2025b; Von Der et al., 2025; Abboud et al., 2023). Consequently, surgical treatment for pyogenic spondylodiscitis has become increasingly common across Europe.

Despite advances in surgical techniques and perioperative management, instrumented spinal stabilization carries a substantial risk of complications. A proportion of patients require reoperation. Revision surgery in this context is most frequently necessitated by construct failure, which may arise from mechanical instability (e.g., screw loosening, implant breakage, loss of correction) or biological causes such as persistent or recurrent infection (Younes et al., 2026a; Valancius et al., 2013; Neuhoff et al., 2024, 2025a; Keric et al., 2017; Schömig et al., 2022; Acharya et al., 2024). These events often manifest clinically as recurrent pain, neurological deterioration, or progressive deformity and require surgical correction (Schatlo et al., 2023; Lin et al., 2012; Jin et al., 2022; Ackshota et al., 2019; Younes et al., 2026b). Reported overall revision rates following surgical treatment of pyogenic spondylodiscitis range from 5% to 15%, exceeding 20% in high-risk populations and in patients with extensive infection or severe bone destruction (Jin et al., 2022; Zawar et al., 2023; Uzumcugil et al.; Younes et al., 2026c). However, while previous studies have primarily reported overall revision rates, data specifically addressing revision surgery due to construct failure and its predictors remain scarce. Klute et al. evaluated 24 patients who underwent 360° fusion of the thoracolumbar spine using expandable vertebral body replacement cages for destructive vertebral osteomyelitis. Six patients (25%) developed construct-related complications, including vertebral body replacement dislocation, material irritation, and screw dislocation, resulting in revision surgery in five cases (Klute et al., 2024). Beyond this small series, evidence regarding the incidence and determinants of construct-related revision remains limited. A better understanding of the factors associated with construct failure is essential to optimize patient selection, refine surgical strategies, and improve postoperative management. Therefore, the present study aimed to identify risk factors associated with revision surgery due to construct failure following instrumented treatment for pyogenic spondylodiscitis of the cervical, thoracic, and lumbar spine.

## 2 Materials and methods

### 2.1 Patients

This retrospective instrumented spinal stabilization in combination with targeted antibiotic therapy. Patients treated conservatively without surgery and those with insufficient clinical documentation cohort study included patients who underwent surgical treatment for pyogenic spondylodiscitis at the University Medical Center Göttingen between 2013 and 2022. All included patients received or follow-up data were excluded from the analysis.

### 2.2 Preoperative assessment and infection management

Preoperative assessment included routine laboratory testing, two sets of blood cultures (aerobic and anaerobic), and computed tomography (CT) together with magnetic resonance imaging (MRI) to evaluate the extent of spondylodiscitis, vertebral bone destruction, epidural abscess formation, and spinal deformity. Osteoporosis was assessed using CT-based Hounsfield unit measurements of the lumbar vertebrae (L1–L4), with osteoporosis defined as a mean attenuation of <110 HU. Formal dual-energy X-ray absorptiometry (DXA) was not routinely available and therefore could not be used as a standardized diagnostic criterion. Intraoperatively, tissue samples were obtained for microbiological culture. In revision cases, explanted implants were additionally subjected to sonication, although this was not routinely performed during the early study period. Empirical antibiotic therapy, in accordance with current guidelines, consisted of vancomycin combined with ceftriaxone or meropenem until pathogen identification or in cases of negative blood cultures. Thereafter, antimicrobial therapy was tailored according to culture and susceptibility results. Antibiotics were generally administered intravenously for two weeks, followed by oral therapy to complete a total treatment duration of six weeks. In selected patients with resistant organisms or complicated infections, treatment was extended to up to 12 weeks following interdisciplinary discussion. Antimicrobial therapy was discontinued after clinical resolution of infection and normalization of inflammatory markers, including C-reactive protein (CRP) and leukocyte count.

### 2.3 Surgical indication and technique

At our institution, early surgical management is the standard treatment strategy for pyogenic spondylodiscitis once the diagnosis has been confirmed clinically, biochemically, and radiologically using CT and MRI. Immediate surgery was performed in patients presenting with neurological deficits. In patients without neurological impairment, surgical intervention was typically carried out within 72 h of diagnosis.

Surgical indications and the operative technique were determined by the extent of vertebral body destruction, spinal instability, and segmental deformity. Circumferential (360°) fusion was performed in patients with severe structural compromise, including >50% vertebral body destruction, pathological fractures, marked kyphotic deformity, or persistent instability requiring anterior column reconstruction. These procedures were preferentially performed in younger patients who were suitable candidates for a staged surgical approach. The first stage consisted of posterior transpedicular instrumentation performed under navigation or robotic guidance. Posterior fixation typically consisted of a short-segment construct (one level above and one level below the affected vertebra), with additional short pedicle screw placement into the infected vertebra whenever feasible, as anterior column reconstruction was planned to restore spinal stability. This was followed by anterior corpectomy with radical debridement. Anterior column reconstruction was achieved using a distractible vertebral body replacement cage (Obelisc™, Ulrich Medical, Ulm, Germany) with adjustable angulation (0°–15°) to restore sagittal alignment.

In contrast, isolated posterior instrumentation was performed in patients without substantial vertebral body destruction, pathological fracture, or significant deformity, in whom adequate spinal stability could be achieved without anterior reconstruction. Percutaneous pedicle screw–rod constructs were implanted under navigation or robotic guidance. In these patients, posterior fixation typically extended one level above and below the affected segment when the vertebral body was intact, and two levels above and below when a mild bone destruction was present, to provide adequate stability in the absence of anterior column reconstruction. However, the final construct length was individualized according to the extent of infection, bone quality, and intraoperative findings.

Patients presenting with neurological deficits and/or radicular symptoms additionally underwent spinal decompression via interlaminar fenestration, hemilaminectomy, or laminectomy, as appropriate. The surgical approach and instrumentation techniques remained consistent throughout the nine-year study period.

### 2.4 Postoperative management and follow-up

Postoperative assessments included a CT scan of the operated area to evaluate surgical outcome during the initial hospital stay. A hard brace was prescribed for 12 weeks after surgery. Clinical and radiological follow-up using MRI took place 8 weeks after surgery. A further clinical evaluation and CT scan were performed at three months and at one year postoperatively.

### 2.5 Outcome measures

**Fig. 1:**
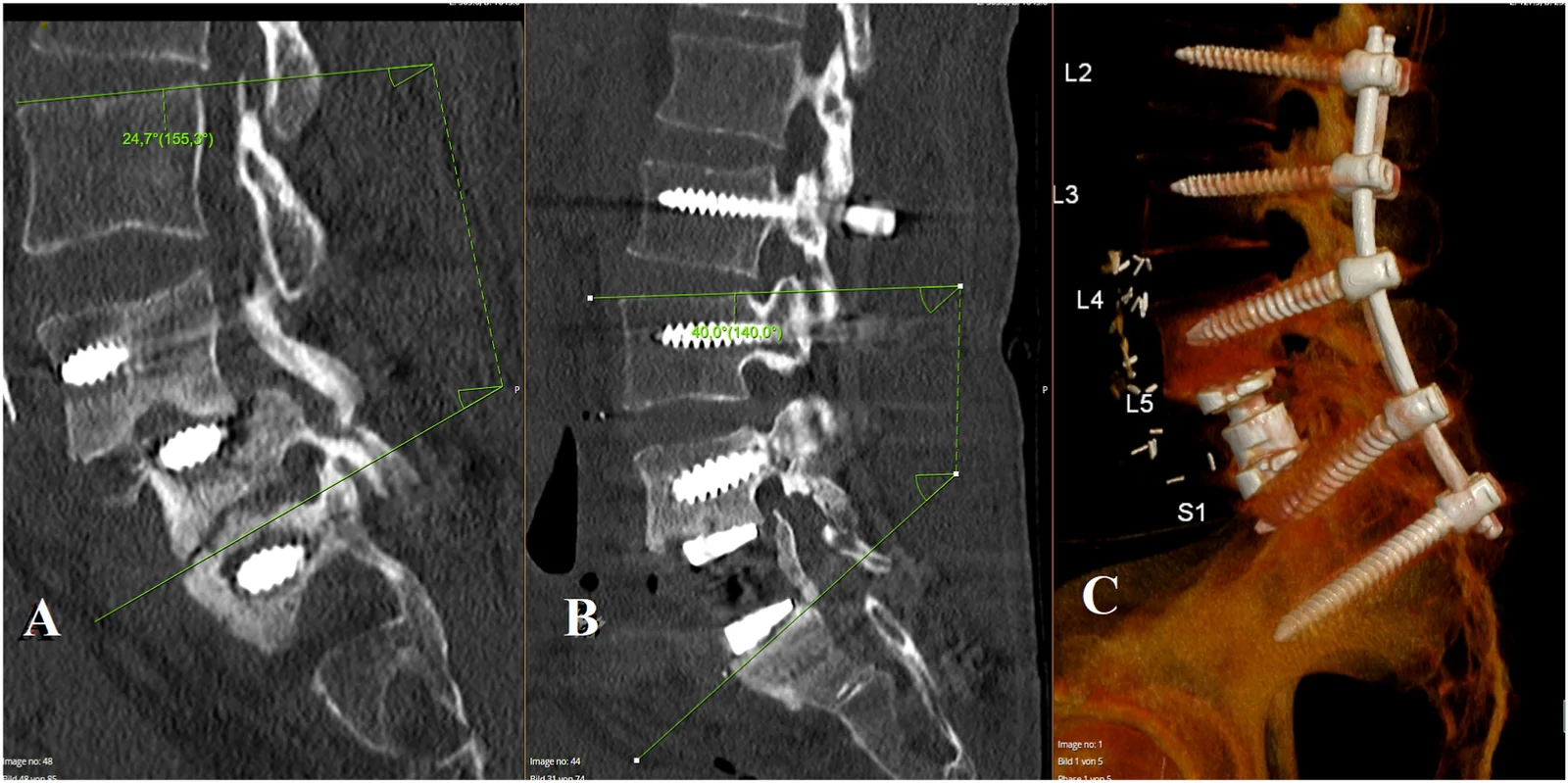
**A:** Patient with construct failure (screw loosening and dislocation after transpedicular instrumentation for spondylodiscitis), Cobb angle is approximately 24°. **B:** Extension of stabilization and corpectomy with cage placement. Cobb angle is now 40°. C: 3D representation after revision surgery.

**Fig. 2:**
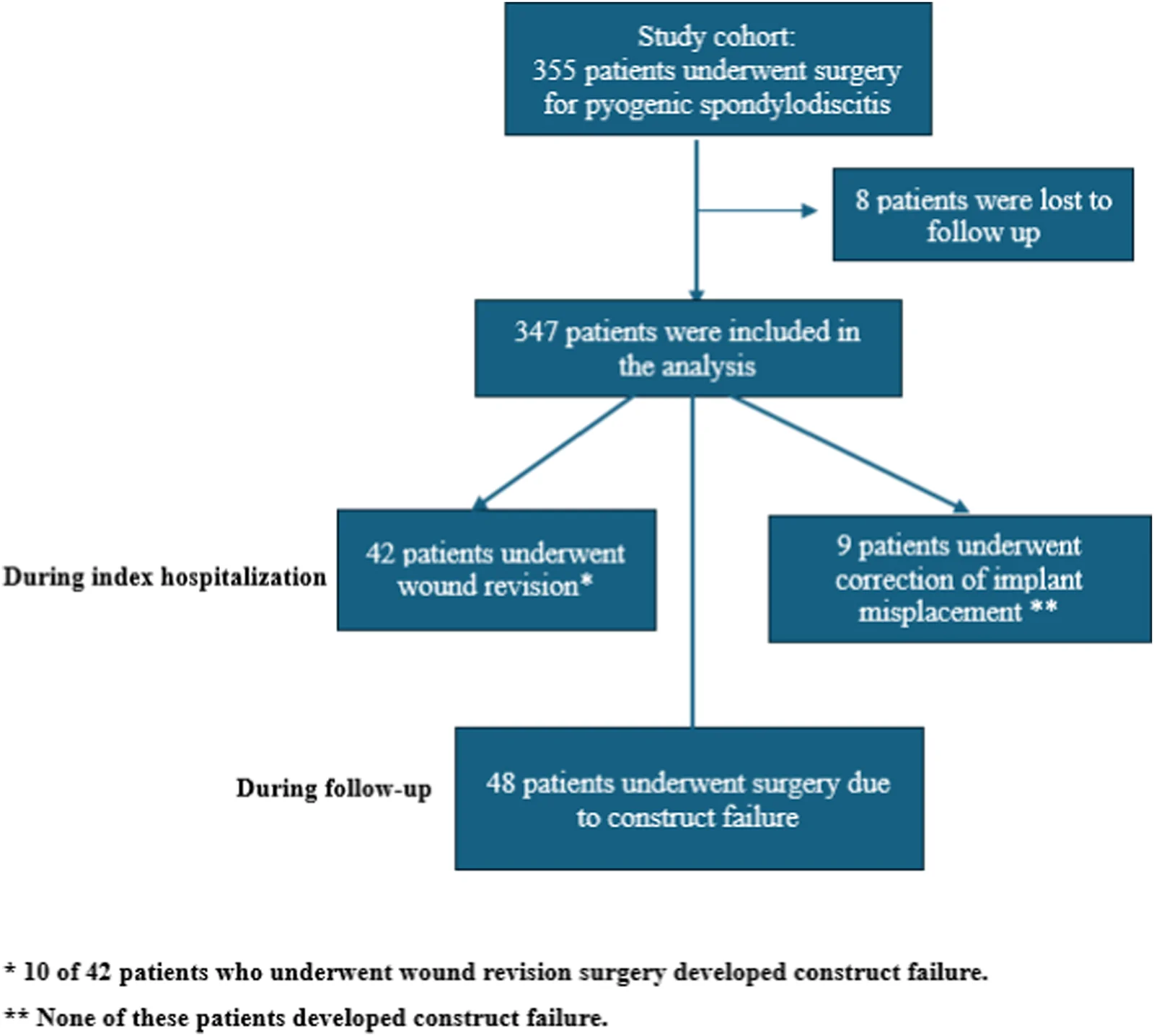
*Patient flowchart showing study enrollment, exclusions, follow-up, and final revision cohort*.

The primary outcome was revision surgery due to construct failure following the index procedure for pyogenic spondylodiscitis. Construct failure included revision surgeries performed for screw loosening, screw or rod breakage, newly developed spinal deformity, pseudoarthrosis, and adjacent segment degeneration (Fig. 1), irrespective of whether these failures were caused by purely mechanical factors or occurred secondary to recurrent infection. Revision surgery was performed in association with recurrent symptoms such as pain and/or neurological deficits. Surgeries performed solely for wound revision or for correction of initial implant misplacement were not classified as construct failure (see Fig. 2).

Relapse of infection was defined as the recurrence of symptoms indicative of pyogenic spondylodiscitis (e.g., back pain or fever) after an initial period of clinical recovery, in combination with radiological evidence on MRI or CT and/or elevated inflammatory laboratory parameters. Relapse infection was analyzed as a potential predictor of revision surgery.

### 2.6 Clinical data and statistical analysis

Data retrieval included patient characteristics as well as intraoperative and postoperative variables potentially associated with infection and revision risk. Data management was performed using Excel database (Microsoft Corp) and IBM SPSS Statistics Version 27.0 (IBM Corp, Released, 2016, IBM SPSS Statistics for Windows, Version 27.0, Armonk, NY, USA).

Time-to-event analyses were performed to evaluate factors associated with the primary outcome. Time was calculated from the date of index surgery to the date of revision surgery due to construct failure. Patients who did not experience the primary outcome were censored either at the time of death or at last follow-up, as appropriate. Kaplan–Meier survival analysis was used to estimate revision-free survival. Cox proportional hazards regression was applied to identify baseline predictors and postoperative factors associated with primary outcome. To avoid model overfitting given the limited number of outcome events, the multivariable Cox regression model was restricted to clinically relevant baseline variables selected a priori based on their availability before surgery, clinical relevance, and biological plausibility. Given the 48 long-term revision events, the number of covariates was further limited in accordance with the commonly accepted recommendation of approximately 10 events per predictor variable.

The baseline Cox model included age, Charlson Comorbidity Component (unadjusted for age), preoperative CRP, and osteoporosis. An extended Cox model was subsequently constructed and adjusted for key baseline confounders (age, Charlson Comorbidity Component, and preoperative CRP), with additional inclusion of postoperative and follow-up variables of interest, namely relapse infection and wound infection. Continuous variables are presented as mean ± standard deviation and were compared using Student's t-test. Categorical variables are reported as counts and percentages and were compared using the chi-square test or Fisher's exact test, as appropriate. A p-value < 0.05 was considered statistically significant.

### 2.7 Ethics statement

Ethical approval was obtained (ethical commission of University Hospital Göttingen, application number: 3/12/17), aligning with the 1964 Declaration of Helsinki and its amendments. All procedures adhered to local and institutional laws and data protection regulations.

## 3 Results

A total of 355 patients who underwent surgical treatment for spondylodiscitis were included in the study. The most common spondylodiscitis location was the lumbar spine found in 200 cases (56%), followed by the thoracic spine in 130 cases (37%) and the cervical spine in 25 cases (7%). The number of affected levels is as follows: one level in 127 cases (36%), two levels in 57 cases (16%), three levels in 60 cases (17%), and more than three levels in 113 cases (32%). The most common pathogen found in either blood or tissue cultures was *Staphylococcus aureus* (50%), *Staphylococcus epidermidis* (9%) and *Escherichia coli* (5%) and negative cultures (29%).

### 3.1 Revision surgery cohort due to construct failure

In this study, 48 patients (13.8%) underwent revision surgery due to construct failure. Baseline characteristics are presented in Table 1, and intraoperative, postoperative, and follow-up events are summarized in Table 2. The most common radiological reason for construct failure was screw dislocation in 36 patients (75%). Additionally, pseudoarthrosis along with degeneration of adjacent spinal segments was noted in 8 patients (17%), new deformities were observed in 12 patients (25%) and hardware breakage occurred in 2 patients (4%). All patients who underwent revision surgery had severe local and/or radicular pain, and the primary goal of the revision surgery was to reduce this complaint. Loosened screws were revised in 28 patients. In 22 of these patients, an alternative screw trajectory could be used, the dorsal instrumentation was extended by two levels above and below the affected segment, whereas in 6 patients the original trajectory was reused with thicker and longer screws and the dorsal instrumentation was extended by three levels cranially and caudally. In 4 cases, reinsertion of screws was not feasible because of severe bone destruction. In 2 patients, all instrumentation was removed, and antibiotic therapy was administered for 12 weeks without subsequent re-instrumentation. In both cases, clinical symptoms improved and no progression of kyphosis was observed following screw explantation. In cases with newly developed spinal deformity (n = 12), dorsal instrumentation was extended by two levels cranially and caudally, combined with corpectomy and placement of an expandable vertebral body cage in 8 patients. In the remaining 4 patients, dorsal transpedicular instrumentation was extended by three levels cranially and caudally without anterior reconstruction. In two cases of rod breakage, dorsal transpedicular instrumentation was extended by three levels above and below the affected segment, and two additional cross-connectors were placed to enhance construct stability.

**Table 1:** Baseline characteristics of patients with and without revision due to construct failure.

Variable	No long-term revision (n = 299)	Surgery of construct failure (n = 48)	Effect size (OR, 95% CI)	P-value
Age (years), mean ± SD	71 ± 12	69 ± 13	—	0.25
Male sex, n (%)	168 (56%)	27 (56%)	1.00 (0.54–1.85)	0.76
Obesity, n (%)	25 (8%)	4 (8%)	1.00 (0.33–3.00)	0.98
Smoking, n (%)	28 (9%)	5 (10%)	1.13 (0.41–3.07)	0.77
Drug abuse, n (%)	15 (5%)	4 (8%)	1.72 (0.55–5.42)	0.36
Hounsfield unit * (mean)	117 ± 55	105 ± 43	-	0.085
MRSA infection, n (%)	29 (10%)	2 (4%)	0.40 (0.09–1.75)	0.17
Antibiotics prior to surgery, n (%)	217 (73%)	31 (65%)	0.69 (0.36–1.31)	0.14
Charlson Comorbidity Index, mean ± SD	8.5 ± 2.0	9.2 ± 3.0	—	0.07
Preoperative CRP (mg/L), mean ± SD	103.3 ± 94.0	117.5 ± 107.0	—	0.403
Cervical involvement, n (%)	22 (7%)	3 (6%)	1.71 (0.61–4.83)	0.46
Thoracic involvement, n (%)	112 (37%)	18 (38%)	1.23 (0.66–2.28)	0.66
Lumbar involvement, n (%)	173 (58%)	27 (56%)	1.26 (0.68–2.37)	0.39

Continuous variables were compared using Student's t-test. Categorical variables were analyzed using the chi-square test or Fisher's exact test, as appropriate. * Osteoporosis was defined as CT-based Hounsfield units <110.

**Table 2:** Intraoperative, postoperative, and follow-up events in patients with and without revision due to construct failure.

Variable	No long-term revision (n = 299)	Surgery of construct failure (n = 48)	Effect size (OR, 95% CI)	P-value
Number of operated levels, mean ± SD	2.5 ± 1.7	2.8 ± 1.4	—	0.20
Monosegmental surgery, n (%)	132 (38%)	13 (27%)	0.61 (0.30–1.22)	0.13
Spinal canal decompression, n (%)	83 (28%)	11 (23%)	0.77 (0.38–1.59)	0.33
Corpectomy/360° fusion, n (%)	50 (17%)	8 (16%)	1.00 (0.44–2.26)	0.66
Hospital stay (days), mean ± SD	22 ± 12	26 ± 12	—	0.015
Wound infection, n (%)	38 (13%)	11 (23%)	2.04 (0.96–4.34)	0.073
Wound revision, n (%)	32 (11%)	10 (21%)	2.20 (0.97–4.98)	0.056
Durotomy, n (%)	6 (2%)	2 (4%)	2.12 (0.42–10.84)	0.66
Relapse infection, n (%)	8 (3%)	14 (29%)	14.98 (5.86–38.29)	<0.001

Intraoperative, postoperative, and follow-up variables are reported for descriptive purposes only. Statistical comparisons were performed using Student's t-test for continuous variables and chi-square or Fisher's exact test for categorical variables.

### 3.2 Revision outcomes

At the 3-month follow-up after revision surgery, 40 of 48 patients (83%) continued to report persistent local and/or radicular pain. At the 6-month follow-up, persistent pain was reported by 35 of 48 patients (73%). Overall, 16 patients ultimately underwent circumferential (360°) fusion. Cage subsidence occurred in 5 of these 16 patients (31%), including two thoracic and three lumbar cases. In four patients, the cages remained stable within the posterior construct and no further intervention was required. One patient underwent additional revision because of cage dislocation associated with progressive vertebral destruction; the corpectomy was extended by one vertebral level and the posterior instrumentation was extended by two additional levels above and below the affected segment. At the 6-month follow-up, screw loosening was observed in 6 of 48 patients (13%). One patient underwent further posterior extension of the instrumentation, one patient underwent circumferential (360°) fusion, three patients underwent isolated removal of the loosened screws, and one patient was managed conservatively because neither neurological deterioration nor radiographic progression was observed. Four patients died within six months after their final revision procedure, three from multiorgan failure and one from pulmonary embolism.

### 3.3 Microbiological findings

In 14 revision cases (29%), all microbiological investigations remained negative during both the initial diagnostic workup and revision surgery despite repeated sampling. In another 14 patients (29%), no pathogen was identified initially; however, cultures obtained during revision surgery yielded positive results. The microorganisms isolated at revision included *Escherichia coli* (n = 3), *Candida albicans* (n = 1), *Klebsiella pneumoniae* (n = 2), *Staphylococcus epidermidis* (n = 2), *Staphylococcus aureus* (n = 4), *Enterococcus faecium* (n = 1), and *Pseudomonas aeruginosa* (n = 1), with polymicrobial infections identified in 4 patients. In 9 patients (19%), the causative microorganism differed between the initial diagnosis and revision surgery, suggesting reinfection or pathogen replacement. Six of these patients initially had *Staphylococcus epidermidis* infection; at revision, cultures yielded *Pseudomonas aeruginosa* in three cases, *Staphylococcus aureus* in two cases, and *Proteus mirabilis* in one case. One patient with an initial *Pseudomonas aeruginosa* infection also had *Staphylococcus aureus* isolated at revision surgery. In the remaining two patients, the initial pathogen was *Enterococcus faecalis*, whereas revision cultures yielded *Staphylococcus aureus* in one patient and *Candida albicans* in the other. In the remaining 11 patients (23%), pathogens identified during the initial diagnostic workup were not detected at revision surgery despite repeated microbiological investigations.

#### 3.3.1 Analyses

**Fig. 3:**
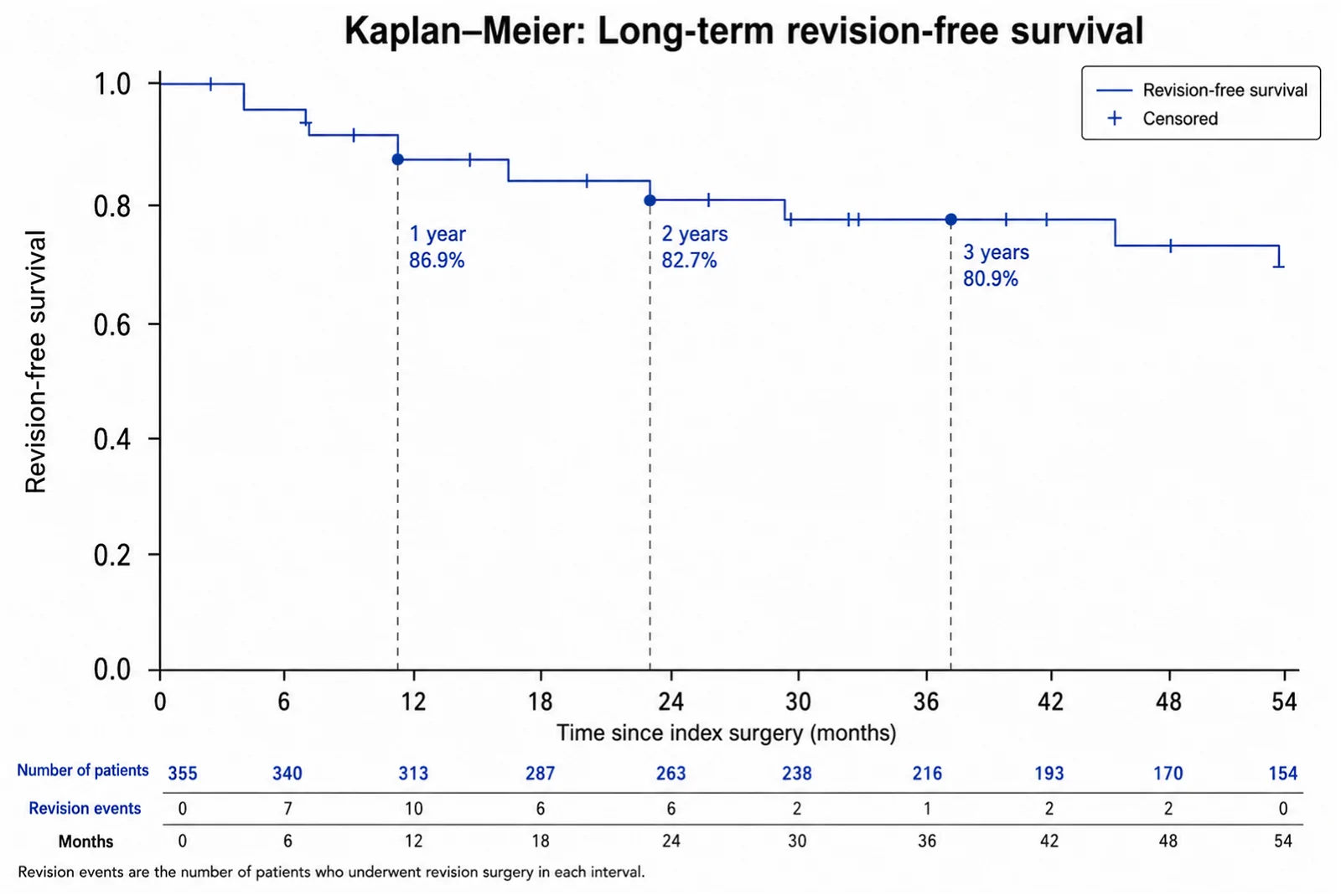
Kaplan–Meier curve illustrating long-term revision–free survival. Time is shown in months from index surgery to long-term revision or censoring (death or last follow-up).

Kaplan–Meier analysis demonstrated revision-free survival rates of 86.9%, 82.7%, and 80.9% at 1, 2, and 3 years, respectively (Fig. 3). Among the 48 patients who underwent revision surgery for construct failure, 12 (25%) required revision within the first 3 months after the index procedure, 20 (42%) between 4 and 12 months postoperatively, and 16 (33%) more than 12 months after the index procedure (up to 48 months of follow-up). The mean time to revision was 10.8 ± 15.6 months. Of these revision surgeries, 3 (6%) involved the cervical spine, 18 (38%) the thoracic spine, and 27 (56%) the lumbar spine. The anatomical distribution of revision surgeries should be interpreted in the context of the baseline distribution of instrumented spinal regions presented in Table 1.

Circumferential (360°) fusion was performed in 50 of 355 patients, with a mean of 4 ± 1.9 instrumented levels, and in 8 of 48 patients in the revision cohort, with a mean of 3.5 ± 1.5 instrumented levels. There was no significant difference in revision rates between groups (Fisher's exact test, p = 0.66).

In the baseline Cox regression model including age, Charlson Comorbidity Component, preoperative CRP, and osteoporosis, a higher Charlson comorbidity burden (HR 1.08 per point increase, 95% CI 1.00–1.16; p = 0.048) and elevated preoperative CRP (HR 1.004 per mg/L increase, 95% CI 1.001–1.007; p = 0.018) were independently associated with an increased hazard of the primary outcome. Age (HR 0.99, 95% CI 0.96–1.02; p = 0.623) and osteoporosis (HR 1.37, 95% CI 0.32–5.89; p = 0.668) were not significantly associated with revision risk (Table 3).

**Table 3:** Cox proportional hazards regression – Baseline model.

Variable	HR	95% CI	p-value
Age (per year)	0.99	0.96–1.02	0.623
Charlson Comorbidity Component (per point)	1.08	1.00–1.16	0.048*
Preoperative CRP (per mg/L)	1.004	1.001–1.007	0.018*
Osteoporosis (yes vs no)	1.37	0.32–5.89	0.668

Baseline Cox model including preoperative variables only (complete-case analysis).

**Fig. 4:**
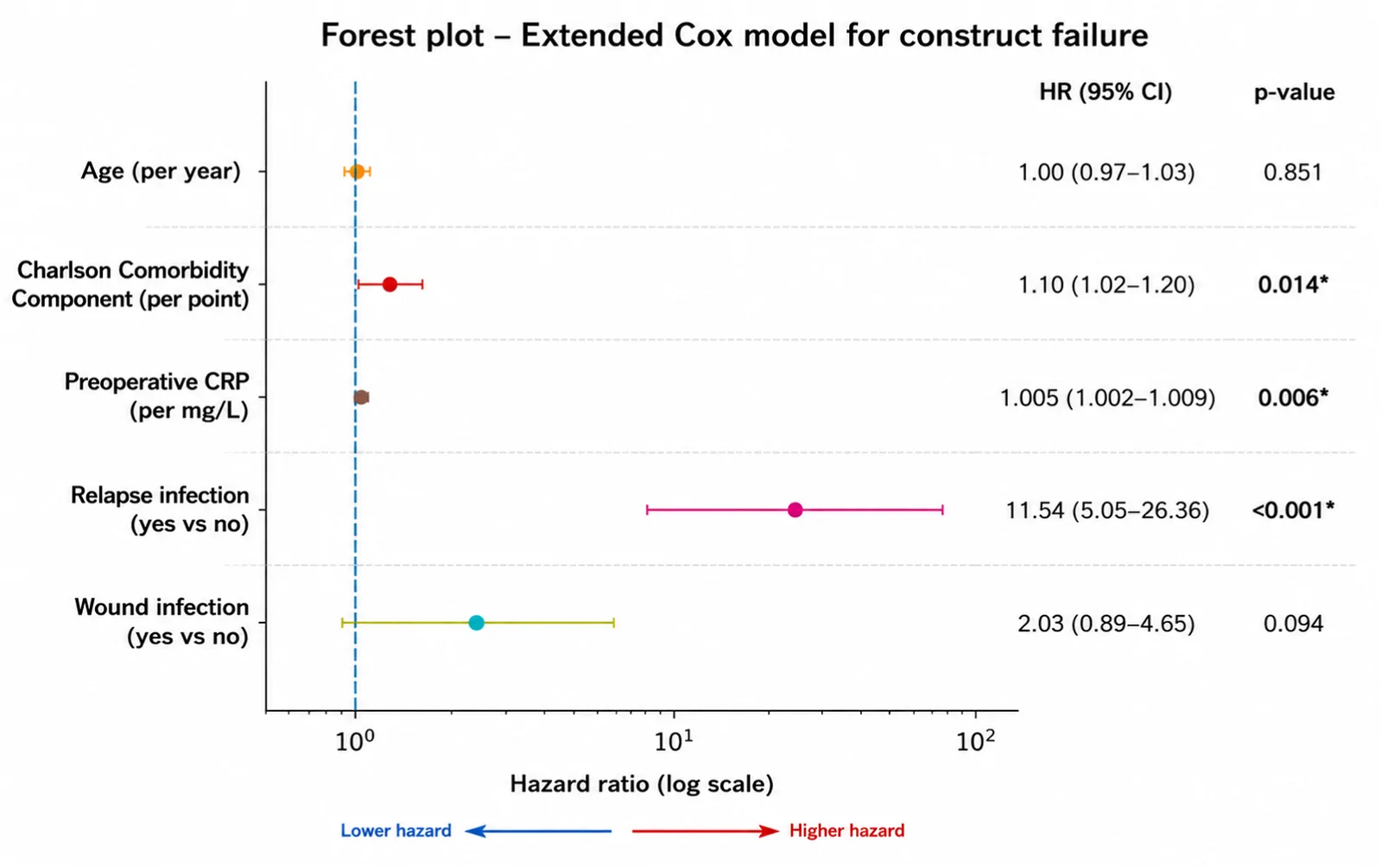
Forest plot showing hazard ratios and 95% confidence intervals from the extended Cox proportional hazards model for long-term revision. The model was adjusted for age, Charlson comorbidity component, and preoperative C-reactive protein, and additionally included relapse infection and wound infection.

In the extended Cox model adjusted for baseline risk factors, relapse infection emerged as the strongest independent predictor of the primary outcome (HR 11.54, 95% CI 5.05–26.36; p < 0.001). Preoperative CRP (HR 1.005 per mg/L increase, 95% CI 1.002–1.009; p = 0.006) and Charlson Comorbidity Component (HR 1.10 per point increase, 95% CI 1.02–1.20; p = 0.014) remained significantly associated with increased revision hazard. Postoperative wound infection was associated with a higher hazard but did not reach statistical significance (HR 2.03, 95% CI 0.89–4.65; p = 0.094) (Table 4, Fig. 4). Sensitivity analyses were performed under extreme-case assumptions to address the eight patients with missing follow-up data. In the worst-case scenario, all eight patients were assumed to have undergone revision surgery due to construct failure, increasing the overall revision rate to 15.8%, whereas in the best-case scenario the revision rate remained 13.8%. Under both assumptions, Kaplan–Meier revision-free survival estimates changed only marginally, and the direction and statistical significance of all Cox regression predictors remained unchanged. Relapse infection consistently remained the strongest independent predictor of long-term revision due to construct failure with hazard ratios remaining above 10 and highly significant (p < 0.001), followed by preoperative CRP and Charlson Comorbidity Component, confirming the robustness of the primary findings.

**Table 4:** Cox proportional hazards regression – Extended model.

Variable	HR	95% CI	p-value
Age (per year)	1.00	0.97–1.03	0.851
Charlson Comorbidity Component (per point)	1.10	1.02–1.20	0.014*
Preoperative CRP (per mg/L)	1.005	1.002–1.009	0.006*
Relapse infection (yes vs no)	11.54	5.05–26.36	<0.001*
Wound infection (yes vs no)	2.03	0.89–4.65	0.094

Extended Cox model adjusted for baseline confounders and including postoperative/follow-up variables.

## 4 Discussion

In this cohort of 355 surgically treated spondylodiscitis patients, revision-free survival remained high, with rates of 80.9% at 3 years. However, 13.8% of patients (n = 48) required revision surgery due to long term construct failure. Neuhoff et al. reported on 31 patients with a follow-up exceeding one year and observed a revision rate due to implant failure of 11%, including posterior pedicle screw loosening (8%) and anterior cage subsidence (3%) (Neuhoff et al., 2024). At our institution, posterior instrumentation alone without discectomy is considered sufficient in most patients to achieve spinal stability and early mobilization, whereas circumferential reconstruction with corpectomy is reserved for severe vertebral body destruction and rigid kyphotic deformity. Although alternative interbody fusion techniques such as XLIF, TLIF, or PLIF may be considered in selected cases, Schatlo et al. reported no significant differences in clinical outcomes according to the surgical technique used for pyogenic spondylodiscitis(Schatlo et al., 2023).

Multivariable Cox regression identified relapse infection as the strongest independent predictor of long-term revision due to construct failure (HR 11.54, 95% CI 5.05–26.36; p < 0.001), while higher Charlson Comorbidity Component scores and elevated preoperative CRP levels were also consistently associated with increased revision risk. Although elevated preoperative CRP was independently associated with an increased risk of revision, the hazard ratio reflects the effect of each 1 mg/L increase and is therefore expected to be modest. Consequently, CRP should not be interpreted as an isolated predictor of construct failure but rather as a surrogate marker of the underlying inflammatory burden. When considered together with clinical findings, comorbidity burden, and evidence of recurrent infection, markedly elevated CRP levels may help identify patients who warrant closer postoperative surveillance.

The relapse of infection can compromise the entire bone structure and is more difficult to treat due to the presence of implants and increased resistance to antibiotics (Schomacher et al., 2014; Fayazi, 2004). It also triggers an ongoing inflammatory response that affects both soft tissue and bone. This inflammatory process can prevent proper healing and interfere with bone remodeling, which is critical for maintaining the stability of spinal implants (Shiban et al., 2020). The presence of a relapse infection often necessitates revision surgeries to remove the infected hardware, debride the infected tissue, and re-stabilize the spine (Schomacher et al., 2014; Fayazi, 2004). This is why it is very important to try to identify the pathogen before starting antibiotic treatment and to ensure that the infection is treated for a sufficient duration using all available diagnostic methods. Shiban et al. reported that screw loosening should raise a high index of suspicion for low-grade infection and demonstrated that sonication of explanted implants significantly increased bacterial yield in both groups (Shiban et al., 2020). In our current practice, all explanted screws are routinely submitted for sonication; however, this was not performed at the outset of the present study. We believe that relapse of infection may be a clinical indicator of an underlying low-grade infection. Sommer et al. documented revision surgery in 20% (14/70) of all patients who underwent surgery for spondylodiscitis. The most common reason for revision was recurrence of spondylodiscitis, followed by screw dislocation, and wound infection (Sommer et al., 2023). Fayazi and Schomacher et at. have reported that pseudarthrosis was observed in approximately 10% of patients following posterior fixation via interbody fusion for pyogenic spondylitis (Schomacher et al., 2014; Fayazi, 2004). A systematic review and meta-analysis on the influence of instrumentation type on outcomes after the surgical management of spondylodiscitis shows no significant differences in fusion rates. Fusion rates were 93.4% with titanium, 98.6% with allograft, 84.2% with autologous bone graft, and 93.9% with polyetheretherketone (PEEK). Screw loosening rates were 0.33% with titanium, 0% with allograft, 1.3% with autologous bone graft, and 8.2% with polyetheretherketone (Maddy et al., 2024). Lin et al. demonstrated that patients with multiple medical comorbidities frequently experience complications, such as infection relapse and pseudarthrosis. The risk of pseudarthrosis may be even higher than reported figures suggest, especially in the later stages of pyogenic spondylitis (Lin et al., 2012). Similarly, in our analysis, higher Charlson Comorbidity Index scores and elevated preoperative CRP levels were consistently associated with an increased risk of revision. No other evaluated variables were independently associated with revision risk. Even though Bettag et at. showed that patients with lower estimated bone mineral density (BMD) have an increased likelihood of requiring revision surgery due to implant failure, it also highlights a possible association between low BMD and implant loosening (Bettag et al., 2020). Osteoporosis was not independently associated with revision risk in our cohort. Postoperative wound infection and wound revision showed borderline associations with long-term revision in our univariate analyses. However, these effects did not reach statistical significance after adjustment and were attenuated in multivariable time-to-event models. This finding likely reflects the close temporal and clinical relationship between early postoperative wound complications and subsequent relapse infection, which emerged as the strongest predictor of long-term revision. Thus, wound infection and wound revision may represent intermediate events along the causal pathway rather than independent risk factors. The observed trends nonetheless suggest that early postoperative wound complications warrant close surveillance, as they may identify patients at increased risk for later revision through persistent or recurrent infection. From a clinical perspective, our findings may facilitate postoperative risk stratification. Patients with relapse infection, a higher Charlson Comorbidity Index, and elevated preoperative CRP levels appear to represent a subgroup at increased risk of construct failure. Accordingly, postoperative surveillance with serial inflammatory marker measurements (e.g., CRP) and CT imaging at approximately 3, 6, and 9 months after surgery may facilitate the early detection of recurrent infection and/or construct failure, particularly in patients with a higher comorbidity burden. However, this surveillance strategy requires prospective validation before it can be considered a standardized follow-up protocol.

### 4.1 Strengths and limitations

The main strength of the present study is the large cohort of patients undergoing instrumented surgery for pyogenic spondylodiscitis and the comprehensive evaluation of predictors associated with revision surgery due to construct failure. Nevertheless, several limitations should be acknowledged. First, the retrospective single-center design introduces the potential for selection bias and limits the generalizability of the findings. Surgical decision-making, including the choice of surgical approach, construct configuration, and timing of revision, was based on individual patient characteristics and surgeon judgment, despite generally consistent institutional treatment principles, and may therefore have introduced heterogeneity in treatment strategies. Second, although osteoporosis was assessed using CT-based Hounsfield unit measurements, standardized assessment of bone quality using dual-energy X-ray absorptiometry was not available for all patients. In addition, detailed radiographic alignment parameters, including sagittal balance, were not consistently documented and therefore could not be incorporated into the analysis. Third, despite adjustment using multivariable Cox regression, residual confounding from unmeasured factors cannot be excluded, particularly with regard to infection severity, pathogen virulence, antimicrobial adherence, nutritional status, and other clinical variables that may influence construct failure. Finally, although the statistical approach was designed to minimize overfitting, the relatively small number of revision events limited the number of variables that could be included in the multivariable model and may have reduced the ability to detect weaker associations. Prospective multicenter studies with standardized clinical and radiographic follow-up are warranted to validate these findings and further refine risk stratification for construct failure.

## 5 Conclusion

Relapse infection was strongly associated with long-term revision surgery due to construct failure following instrumented treatment for pyogenic spondylodiscitis. A higher comorbidity burden and elevated preoperative CRP were also associated with an increased risk of revision. These findings may help identify patients at increased risk of construct failure who could benefit from closer postoperative surveillance. However, prospective studies are required to validate these associations and determine whether targeted interventions can improve clinical outcomes.

## Ethics approval and consent to participate:

This retrospective chart review study involving human participants was in accordance with the ethical standards of the institutional and national research committee and with the 1964 Helsinki Declaration and its later amendments or comparable ethical standards. The Human Investigation Committee (IRB) of University Hospital Göttingen, application number: 3/12/17 approved this study. All procedures adhered to local and institutional laws and data protection regulations. Informed consent was not required as the study was retrospective in nature and involved the analysis of previously collected data.

## Clinical trial number:

Not applicable.

## Availability of data and materials

The datasets used and/or analyzed during the current study are available from the corresponding author on reasonable request.

## Consent for publication:

Not applicable.

For this retrospective study, formal consent for publication was not required, as all data were anonymized and collected in accordance with institutional ethical standards and national data protection regulations.

## Authors' contributions

Tammam Abboud: made the figure and corrected the manuscript. Bilal Younes: designed the project, wrote the manuscript, made the figure and analyzed data. Charlotte Flüh: provided scientific support. Dorothee Mielke: provided scientific support and corrected the manuscript. Veit Rohde: provided scientific support and corrected the manuscript. All authors fulfil the criteria for authorship of the International Committee of Medical Journal Editors.

## Funding:

The authors declare that no funds, grants, or other financial support were received during the preparation of this manuscript.

## Declaration of competing interests

The authors report no conflict of interest concerning the materials or methods used in this study or the findings specified in this paper.
